# Detection and Mitigation of SYN Flooding Attacks through SYN/ACK Packets and Black/White Lists

**DOI:** 10.3390/s23083817

**Published:** 2023-04-07

**Authors:** Chun-Hao Yang, Jhen-Ping Wu, Fang-Yi Lee, Ting-Yu Lin, Meng-Hsun Tsai

**Affiliations:** 1 Department of Computer Science and Information Engineering, National Cheng Kung University, Tainan 701401, Taiwan; 2 Department of Computer Science, National Yang Ming Chiao Tung University, Hsinchu 300093, Taiwan

**Keywords:** software-defined network (SDN), programmable data plane, cybersecurity, SYN flooding

## Abstract

Software-defined networking (SDN) is a new network architecture that provides programmable networks, more efficient network management, and centralized control than traditional networks. The TCP SYN flooding attack is one of the most aggressive network attacks that can seriously degrade network performance. This paper proposes detection and mitigation modules against SYN flooding attacks in SDN. We combine those modules, which have evolved from the cuckoo hashing method and innovative whitelist, to get better performance compared to current methods Our approach reduces the traffic through the switch and improves detection accuracy, also the required register size is reduced by half for the same accuracy.

## 1. Introduction

With the development of the Internet, many networking technologies have been developed, including the Internet of Things [1] and software-defined networks (SDN) [2]. However, these online systems suffer from cybersecurity threats that degrade their performance. Therefore, how to detect and mitigate these risks has become a critical issue. In 2020, Bouyeddou et al. [3] presented the most popular denial-of-service (DoS) and distributed DoS (DDoS) attacks: the TCP SYN flooding, UDP flood, Smurf, and ICMPv6-based flooding attacks. The SYN flooding attack is the most aggressive network security attack, which abuses the three-way TCP handshake to rapidly fill the server’s memory storage [4,5]. In traditional networks, SYN flooding has been mitigated by deploying expensive firewalls in front of critical servers. Fortunately, software-defined networking has introduced new ways to mitigate SYN flooding, primarily based on OpenFlow and P4, which provide the standardized interface between the control and data planes.

To reduce the load on the controller and the possibility of saturation attacks, Programming Protocol-independent Packet Processors (P4) was proposed in 2014 [6]. This allows the switches to perform some of the control plane tasks, giving network administrators more flexibility to monitor the network and reducing the possibility of controller-switch overload. Therefore, P4 is adopted in this paper to implement the proposed scheme.

The proposed scheme is based on the combination of the cuckoo hashing [7] and TCP reset [8] methods. First, we set up the detection module by designing a data structure based on cuckoo hashing without kick action. Instead of the kick action, we design a whitelist and a blacklist to block the attackers. The reason for modifying the cuckoo method’s kicking action is that P4 does not support for-loop. Furthermore, the original TCP reset method only adds the client’s IP address to the whitelist table. In our research, we create two tables for the whitelist and the blacklist, recording the source MAC address instead of the source IP address because attackers often change their source IP addresses. With the detection engine and the blacklist, we can block the attacker instead of having the switch return the SYN/ACK packet as in the TCP reset method. The contributions of this paper are summarized as follows.

- The method proposed in this paper reduces about half of the malicious traffic, improves detection accuracy by 2%, and reduces the usage of registers by half compared to existing methods.
- To detect and mitigate more malicious traffic compared with the TCP reset method, which only records IP addresses in a whitelist, our proposed method adds a blacklist and records MAC addresses instead to prevent attackers from changing IP addresses too often and being detected.
- The proposed method is implemented on bmv2 P4 software switch by combining revised cuckoo hashing, a detection module, a and mitigation module. The experimental results prove that it is more advantageous than the existing methods.

This paper is organized as follows. Section 2 introduces some typical cybersecurity attack methods, and some detection/mitigation modules based on OpenFlow and P4 and their drawbacks. Section 3 presents our method for SYN flooding attacks from the evolved combination of cuckoo hashing and TCP reset method. Section 4 shows the performance comparison of our method with the counting Bloom filter and TCP reset method through simulation experiments. Finally, Section 5 illustrates our breakthrough and future work.

## 2. Related Works

In this section, we introduce some common influences on cybersecurity attacks and then take a closer look at several detection and mitigation approaches based on OpenFlow and P4. We also discuss the limitations of these approaches, which motivate us to propose our method.

Cybersecurity threats have become more prevalent since the proliferation of mobile devices and applications [9]. Almaiah et al. [10] realized that classification is an important step before solving the problems.

In the following subsections, we introduce some methods to solve the problems of emerging cybersecurity attacks.

### 2.1. SYN Flooding Attack

TCP Reset [8] protects legitimate packets and establishes connections with benign clients using an authentication mechanism in the absence of any switch-controller communication. When an invalid packet passes through the switch, the switch converts the SYN packet into a SYN/ACK packet. After receiving an ACK packet, it validates it and changes it to an RST packet. If a SYN flood attack occurs in TCP reset, the attacker’s packets should not be responded to at this time. However, to verify that the attacker is a normal client, it still responds to the attacker’s SYN/ACK packets, which means that this process increases unnecessary network traffic.

SAFETY [11] sets the dynamic threshold by calculating Shannon’s entropy [12]. However, since all TCP packets use the controller (packet-in) to collect traffic, it increases the load between the controller and the switch. SLICOTS [13] reduces packet ingress frequency by installing temporary forwarding rules. However, the load between the controller and the switch still increases as traffic increases.

To detect the flooding, Malik et al. [14] presented a Flooding Factor based Framework for Trust Management (F3TM) by using the calculated trust value as the identification for malicious nodes. Alternatively, Sunil et al. [15] used the delimitated anti-jammer scheme to identify a vehicle’s location by establishing vehicle-to-vehicle communication and detecting anomalies in the data, which they also eliminate using the combined function of the foster rationalizer and the morsel supple filter, respectively.

Paolucci et al. [16] assigned two registers to each IP match table to store data using a P4-based method. While the number of connections increases, the switch needs more registers, so packets above the threshold are discarded when the number of attempts exceeds the threshold.

In some studies [4,17], the counting Bloom filter (CBF) [18] is used to collect traffic information at the data plane. Given a counting array and several hash functions, the inputs access the corresponding counters according to the output of the hash functions. However, this method has a drawback in some cases. If the packet rate of the attacker and the normal user are similar, it will result in the same counter for both, so it is not possible to correctly determine the normal user or the attacker, and there may be confusion between them.

### 2.2. Attack to Encryption and Blockchain

As data exchange is rapidly increasing due to the boom in mobile networks, the security of encryption and blockchain is also critical. To prevent attacks and theft, encryption is the most suitable method to protect information from hackers.

A novel hybrid encryption approach between elliptic curve cryptosystem and hill cipher (ECCHC) is proposed, which generates a new encryption/decryption key without sharing the key over the Internet. It prevents attacks by intruders and provides a better security environment for data exchange [19]. Furthermore, Aitizaz et al.  [20] presented a mechanism that allows blockchain users to encrypt data on their side and upload it to the distributed ledger for record purposes.

As we have seen, the possibilities for security, trustworthiness, reliability, and confidentiality are becoming increasingly apparent [20].

## 3. Proposed Scheme

**Figure 1:**
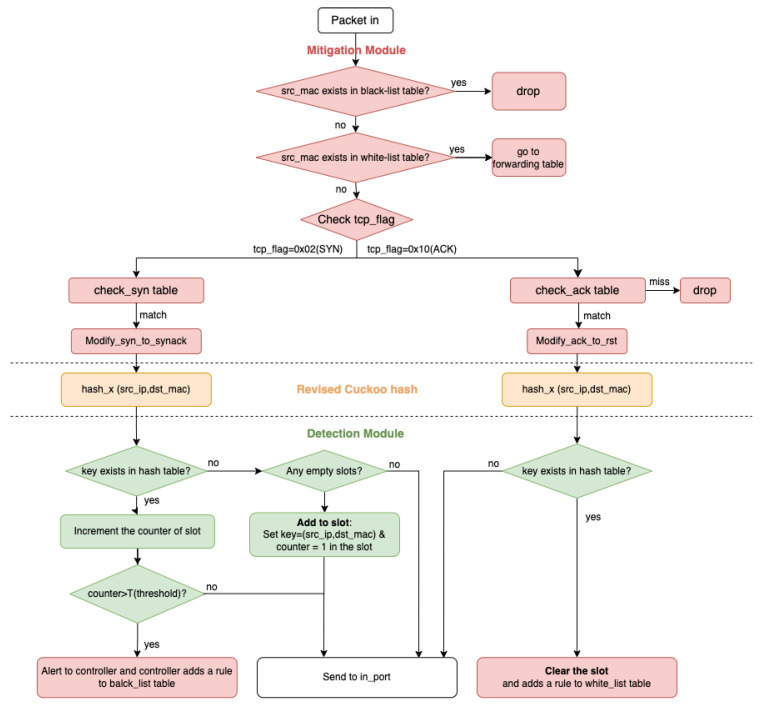
Workflow of our method.

Figure 1 is the workflow of our method. The detection and mitigation modules connect perfectly to our revised cuckoo hash function. Furthermore, in our mitigation module, which is the combination of cuckoo hashing and TCP reset, the detection module produces whitelist and blacklist tables. At the very beginning, the whitelist and blacklist are empty, but after going through the workflow more, these lists become more complete and gain a powerful mitigation capability by constantly updating the whitelist and blacklist, which can drop the suspicious packets as soon as they are received.

### 3.1. Framework of the Proposed Method

This subsection introduces the framework of the method proposed in this paper, which contains the revised cuckoo hashing, the mitigation module, and the detection module. When the packet arrives, it first encounters the mitigation module, which contains the whitelist and the blacklist, followed by the detection module. The packet will be blocked if the source MAC address (src_mac) of the packet exists in the blacklist table. If not, the source MAC address (src_mac) of the packet is checked against the whitelist table. If it matches, the packet goes to the forwarding table. The module distinguishes whether the packet is a SYN or ACK packet if it does not exist in the whitelist and blacklist tables. Then it sends SYN to the check-syn table and ACK to the check-ack table.

After the SYN packet matches an entry of the check-syn table, which has 256 entries, the program will modify the SYN packet into a SYN-ACK packet by using the modify_syn_to_synack action and detecting attackers. Similarly, after the ACK packet matches an entry of the check-ack table, the program will modify ACK to a RESET packet using the modify_ack_to_reset action and detecting attackers.

Note that if the ACK packet does not match an entry of the check-ack table, it means that we have not received the SYN packet from this MAC address before, so the module will drop this suspicious ACK packet.

### 3.2. Revised Cuckoo Hashing

Since the detection is handled by checking whether the key exists in the hash table or not, we introduce a hashing method evolved from cuckoo hashing, called revised cuckoo hashing. Because we use P4 as our approach, where for-loop is not supported, the kicking action cannot be performed until an empty slot is found. Instead, we will clear the slot after classifying the source MAC address to the whitelist and blacklist.

**Figure 2:**
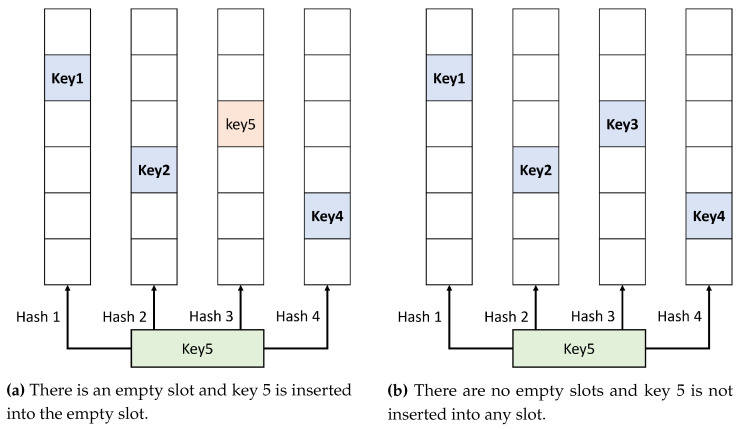
Our hashing scheme.

Figure 2 shows our hashing scheme with four buckets and hash functions. Since we are going to compare our method with the performance of the Bloom filter in our experiments, we set the number of a hash function to four to optimize the performance of the Bloom filter based on [4], which compares the accuracy of the Bloom filter with a varying number of hash functions.

If there is an empty slot, the key is inserted into the empty slot, as shown in Figure 2a. If there is more than one empty slot, the key inserts into the first one it meets. As in Figure 2b, the key does not insert into any slot as long as there is no empty slot.

### 3.3. Detection Module

There is a hash table for storing values in registers, and a slot in the table contains a key and a value. The key stores two fields, the IP and MAC address, and the value stores a counter. The detection module has two main actions: “Add to slot” and “Clear the slot”.

#### 3.3.1. Add to a Slot

Using the source IP address and destination MAC address of the packet as the key, then calculate *k* using different hash functions and obtain the *k* corresponding to the index.

If the key does not exist in the hash table, check if there is still an empty *k* slot, if there is no empty slot, the packet will be sent back to the client; otherwise, the key (source IP address, destination MAC address) will be stored and the key and counter will be set to 1.

Furthermore, if the key exists in the hash table, the counter is incremented by 1 and checked to confirm if the counter is greater than the threshold (*T*). The threshold means that *T* of connection failures are acceptable. If the counter exceeds the threshold, the switch will send summary information to the controller, and then the controller will add the MAC address of the key to the blacklist table. The procedure is shown in Algorithm 1. The time complexity is given in Theorem 1.

**Theorem** **1.** *The time complexity of Algorithm 1 is O(k). The parameter k in Algorithm 1 is the size of the array rand.*

**Proof of Theorem 1.**  In the beginning, the required parameters are read in and the operation is O(1) (lines 1–3). In the for-loop, since *k* represents the size of the array rand, the loop executes *k* times, so the time complexity of this loop is O(k) (lines 4–21). Inside the loop, the hash computation, judgment, and setting are performed in O(1) (lines 5–20). The final judgment on the flag is O(1) (lines 22–24). Therefore, the time complexity of Algorithm 1 is O(k).    □

**Algorithm 1** Add to a slot**Require:** a hash table with *N* slots, hash_table; each slot contains three fields: ip, mac, counter; an array with *k* random numbers, rand; the length of a bucket, row_length; the threshold, *T*;1: **procedure** ADD-A-SLOT(src_ip, dst_mac)2:  now_column, empty_flag, empty_index, find_flag, hash_value←03:  $insert\_slot\leftarrow \left\{ip:\;src\_ip,\;mac:\;dst\_mac,\;counter:\;1\right\}$4:  **for** i = 1 to *k* **do**5:    $hash\_value\leftarrow hash(src\_ip,\;dst\_mac,\;rand\left[i\right])$6:    now_index←now_column∗row_length+hash_value7:    now_column←now_column+18:    read_slot←read_hash_table(now_index)9:    **if** empty_flag=0 and read_slot.counter=0 **then**10:       empty_flag←111:       empty_index←now_index12:    **else if** read_slot.ip=src_ip and read_slot.mac=dst_mac **then**13:       insert_slot.counter←read_slot.counter+114:       **if** insert_slot.counter≥T **then**15:         alert_to_controller(dst_mac)16:         $insert\_slot\leftarrow \left\{ip:\;0,\;mac:0,\;counter:\;0\right\}$17:       **end if**18:       write_hash_table(now_index, insert_slot)19:       find_flag←120:    **end if**21:  **end for**22:  **if** find_flag=0 and empty_flag=1 **then**23:    write_hash_table(empty_index, insert_slot)24:  **end if**25: **end procedure**


#### 3.3.2. Clear the Slot

The program will obtain the number of *k* slots that can be accessed using the key of the input item. Then it will check if the key is in the hash table. The process is shown in Algorithm 2. The time complexity is given in Theorem 2.
**Algorithm 2** Clear the slot**Require:** a hash table with *N* slots, hash_table; each slot contains three fields: ip, mac, counter; an array with *k* random numbers, rand; the length of a bucket, row_length;1: **procedure** CLEAR-SLOT(src_ip, dst_mac)2:  now_column, hash_value←03:  $insert\_slot\leftarrow \left\{ip:\;0,\;mac:\;0,\;counter:\;0\right\}$4:  **for** i = 1 to *k* **do**5:    $hash\_value\leftarrow hash(src\_ip,\;dst\_mac,\;rand\left[i\right])$6:    now_index←now_column∗row_length+hash_value7:    now_column←now_column+18:    read_slot←read_hash_table(now_index)9:    **if** read_slot.ip=src_ip and read_slot.mac=dst_mac **then**10:       write_hash_table(now_index, insert_slot)11:    **end if**12:  **end for**13: **end procedure**

**Theorem** **2.** *The time complexity of Algorithm 2. The time complexity is O(k), where the parameter k is the size of the array rand.*

**Proof of Theorem 2.**  First, Algorithm 2 sets some variables to 0 in O(1) operations (lines 1–3). The for-loop executes *k* times, so the time complexity is O(k) (lines 4–12). Inside the for-loop, the hash operations such as computing, judging, reading, writing, and setting values are all O(1) operations (lines 5–10). Therefore, the time complexity of Algorithm 2 is O(k). □

### 3.4. Mitigation Module

In this paper, the switch is placed near the client and a blacklist table is added to record the attackers after combining the cuckoo hashing and TCP reset methods to prevent the attackers. This blacklist means that the attacker can be prevented from attacking by storing the client’s MAC address. Attackers often change source IPs, so we choose to store MAC addresses instead of IP addresses.

The method proposed in this paper contains five tables, and the meaning represented by each table is as follows.

1. blacklist table: Block the MAC address of the attacker.
2. whitelist table: Check the source MAC address of the packet. If there is a matching entry, the packet will be forwarded. Otherwise, the SYN packet will be put into the check-syn table, the ACK packet will be put into the check-ack table, and the rest of the packet will be dropped.
3. forwarding table: Forward the packets to the corresponding output port.
4. check-syn table: If a packet matches one of the 256 entries, modify_syn_to_synack is executed and the attacker is detected.
5. check-ack table: Verify the ACK number of the packet. If the ACK number is correct, then run modify_ack_to_rst and detect the attacker. Otherwise, the packet is discarded.

## 4. Performance Evaluation

In this section, we introduce the simulation environment and then perform experiments. In comparison with the existing methods, we present the progress and advantages of the proposed method in this paper.

### 4.1. Simulation Environment

**Figure 3:**
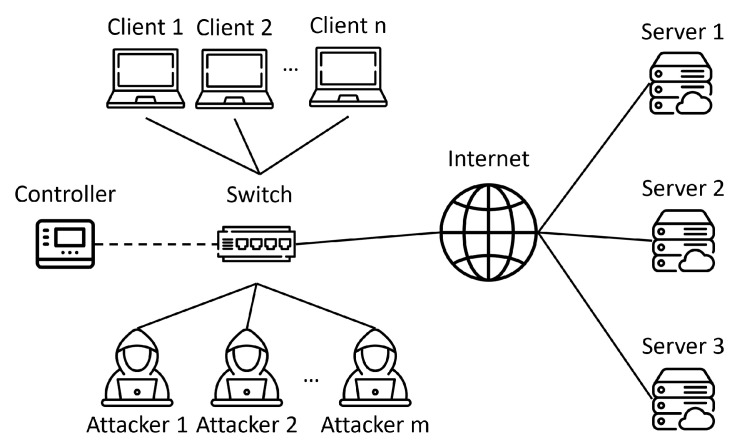
Topology.

Figure 3 shows the experimental topology, which contains *n* normal users and *m* attackers, and the packets sent and received by the host will pass through the switch. Three servers are set up to implement load balancing in the experiment, and the topology is simulated in Mininet [21]. The software switch uses behavioral model version 2 (bmv2) [22,23], which can be compiled for P4. We use curl [24] to establish normal client connections and hping3 [25] for the attackers to perform SYN flooding attacks.

### 4.2. Experiments

In this subsection, we discuss five experiments:

- Experiment 1: Compare the traffic amount of our method and TCP reset.
- Experiment 2: Set the thresholds for the Bloom filter method.
- Experiment 3 and 4: Compare the detection accuracy of our method and the Bloom filter method in the high/low-rate attack and normal user.
- Experiment 5: Compare the required register size of our method and the Bloom filter method in the same situation.

#### 4.2.1. Experiment 1: Compare the Traffic Amount of Our Method and TCP Reset

In a SYN flooding attack, the attacker’s packet rate is typically much higher than a normal user’s. By sending a large number of fake SYN packets, the attacker floods the target host, making it unable to handle normal connection requests. To simulate this scenario, we set the packet rate of 50 normal users to 0.25 (f/s) in our experiment, while the attacker’s packet rate was set to 5 (f/s) for a duration of 30 s.

**Figure 4:**
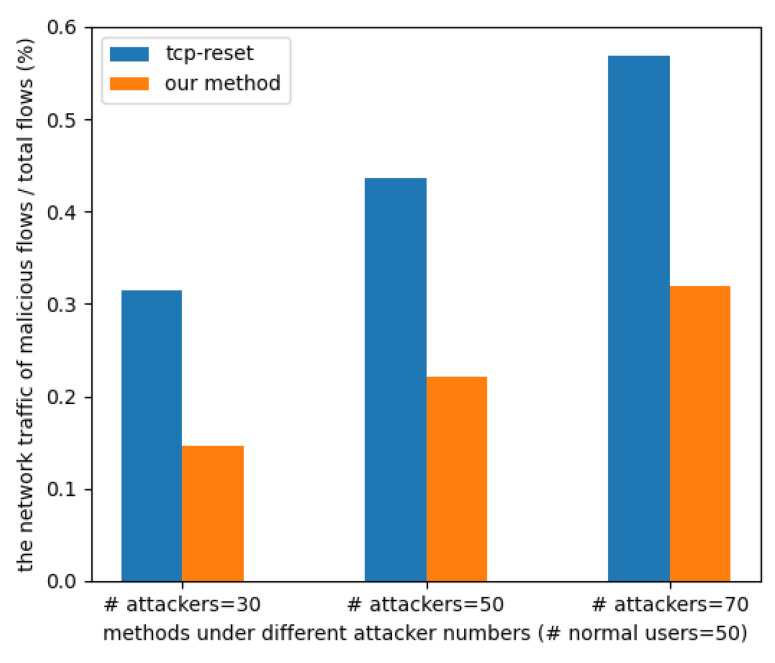
Experiment 1: The percentage of malicious traffic under different attacker numbers.

In Figure 4, we can see the percentage of malicious traffic in the network traffic passing through the switch. TCP reset sends SYN/ACK packets even after receiving malicious SYN packets. The method proposed in this paper can stop sending SYN/ACK packets by adding its MAC address to the blacklist when an attacker is detected. As a result, the percentage of malicious traffic is reduced to about half of the original TCP reset after detection by the method proposed in this paper.

#### 4.2.2. Experiment 2: Set the Thresholds for the Bloom Filter Method

Since the Bloom filter requires predefined thresholds to distinguish between normal users and attackers, the experiment is designed to determine the detection accuracy under different thresholds and the highest accuracy threshold.

**Figure 5:**
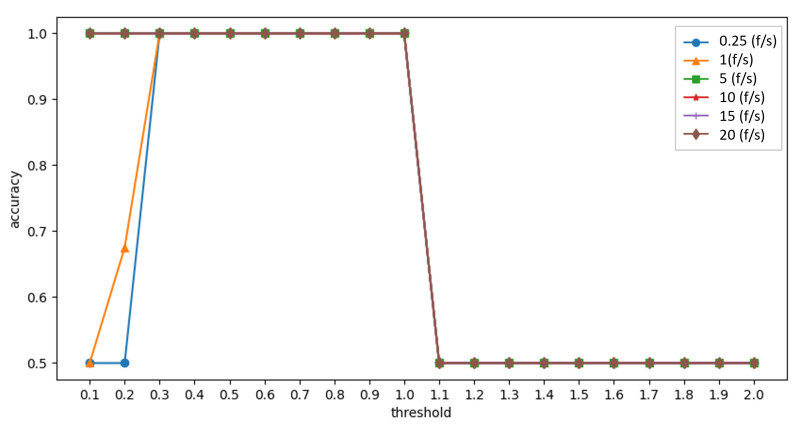
Experiment 2: Effect of thresholds on accuracy.

This experiment has 20 normal users and 20 attackers. The packet rate is 0.25 (f/s) for normal users and 0.25–20 (f/s) for attackers, and the experiment lasts one minute. The experimental results are shown in Figure 5. When the threshold is between 0.3 and 1, the accuracy is close to 100%, so the threshold is set to 0.65, the middle value of the best range between 0.3 and 1.

#### 4.2.3. Experiments 3 and 4: Compare the Detection Accuracy of thE Proposed Method with the Bloom Filter Method for High- and Low-Rate Attacks

This paper focuses on detecting and mitigating SYN flooding attacks, which are high-rate packet attacks of DoS. The other type of DoS attack is a low-rate attack [26], which is not the main issue of this paper, but we hope to prove that our method is also effective under low-rate attacks through experiments. Therefore, in our experiments, we perform experiments on high-rate and low-rate attacks to compare the effectiveness of the Bloom filter and the proposed method in detecting malicious traffic.

##### High-Rate Attack

In this subsection, the number of normal users is first fixed, and the detection accuracy is tested when the number of attackers changes. Then, the normal users are exchanged with the attackers, i.e., the number of attackers is fixed, and the detection accuracy is tested when the number of normal users changes.

**Figure 6:**
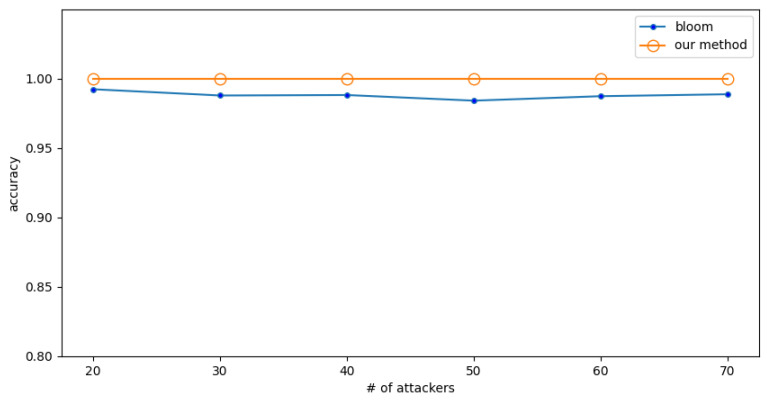
Experiment 3: The effect of numbers of attackers on accuracy under high-rate attack.

**Figure 7:**
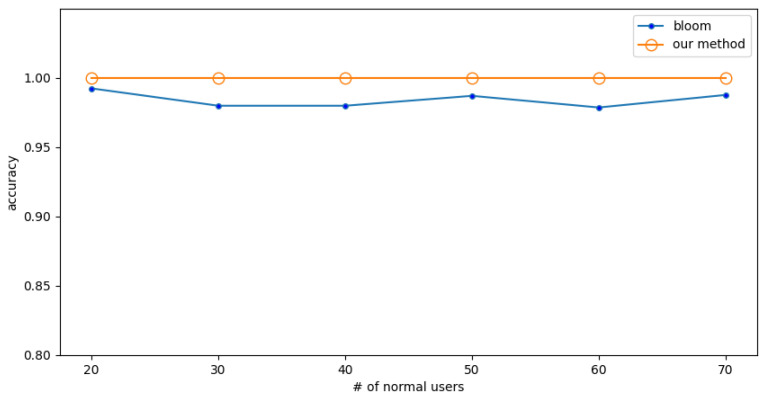
Experiment 3: The effect of numbers of normal users on accuracy under high-rate attack.

In a high-rate attack, the attacker’s packet rate is 5 (f/s), while the normal user’s packet rate is 0.25 (f/s). The detection accuracy when the number of normal users is fixed at 20 and a different numbers of attackers are encountered is shown in Figure 6. The detection accuracy when the number of attackers is fixed to 20 and the number of normal users are different is shown in Figure 7.

The experimental results show that the Bloom filter has some fluctuations, and its accuracy is lower. In comparison, the method proposed in this paper has almost no fluctuations and higher accuracy, which shows that the proposed method is more precise and reliable. The results also show that the accuracy of the proposed method is 2% higher than the Bloom filter.

##### Low-Rate Attack

As in the previous subsection, first fix the number of normal users and experiment with the detection accuracy when the number of attackers changes. Then, the normal users are exchanged with the attackers.

**Figure 8:**
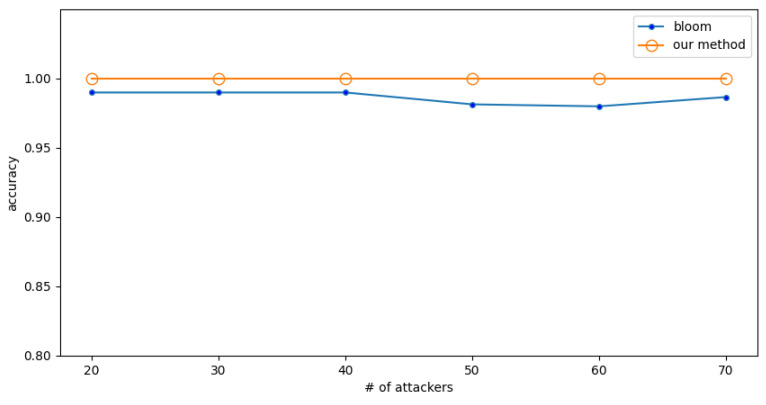
Experiment 4: The effect of numbers of attackers on accuracy under low-rate attack.

**Figure 9:**
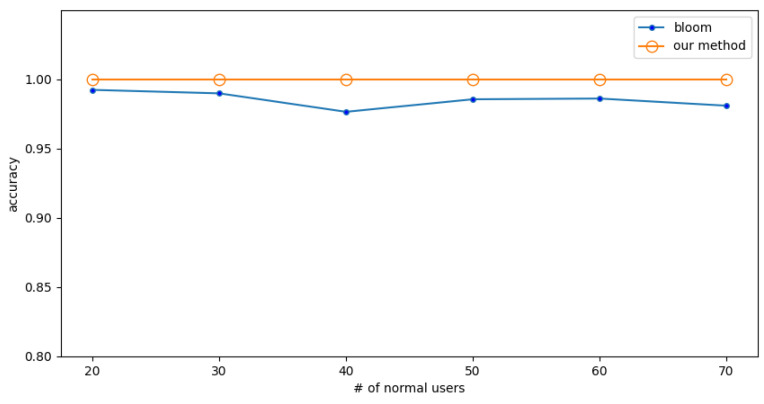
Experiment 4: The effect of numbers of normal users on accuracy under low-rate attack.

In the low-rate attack, the packet rate of normal users and attackers is 0.25 (f/s). The detection accuracy is shown in Figure 8 when the number of normal users is fixed at 20 and the different numbers of attackers are encountered. The detection accuracy is shown in Figure 9 when the number of attackers is fixed to 20 and different numbers of normal users are encountered.

The experimental results show that the Bloom filter has some fluctuations, and its accuracy is lower. In comparison, the method proposed in this paper has almost no fluctuations and higher accuracy, which shows that the proposed method is more precise and reliable. The results also show that the accuracy of the proposed method is 2% higher than the Bloom filter.

#### 4.2.4. Experiment 5: Evaluate the Required Size of Register

Because of the limited size of the registers, it is important to understand the consumption of the registers when it is desirable to use fewer resources. We have therefore designed the following experiment to compare the size of the registers required by the method proposed in this paper with the Bloom filter for different numbers of flows.

Since Mininet cannot generate many hosts, this paper designs its own program to simulate traffic and test how many registers are needed. Assume the slot size is 12 bytes (IP address: 4 bytes; MAC address: 6 bytes; counter: 2 bytes). We create *n* malicious flows using Poisson distribution with a duration of 60 s. In this distribution, λ represents the average of the attacker’s packet rate (f/s). If all attackers are detected within 60 s of the experiment and added to a blacklist with *m* slots, the required register size is m∗12 (bytes).

**Figure 10:**
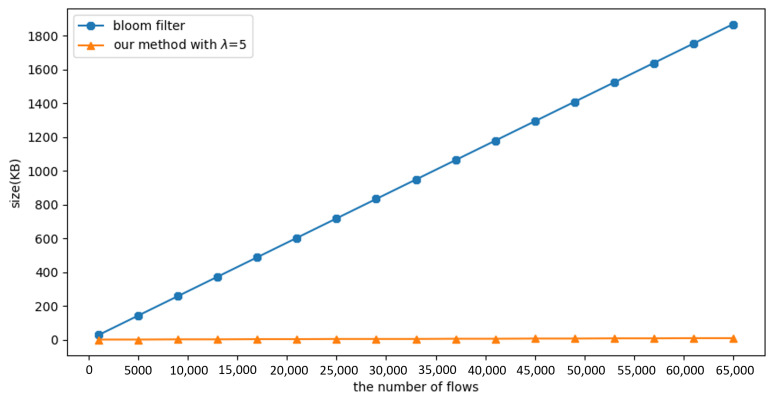
Experiment 5: The required size of the register under different flow numbers.

Figure 10 shows that the Bloom filter requires twice the register size of the proposed method when the flow reaches 65,535, i.e., the proposed method consumes only half the register usage.

**Figure 11:**
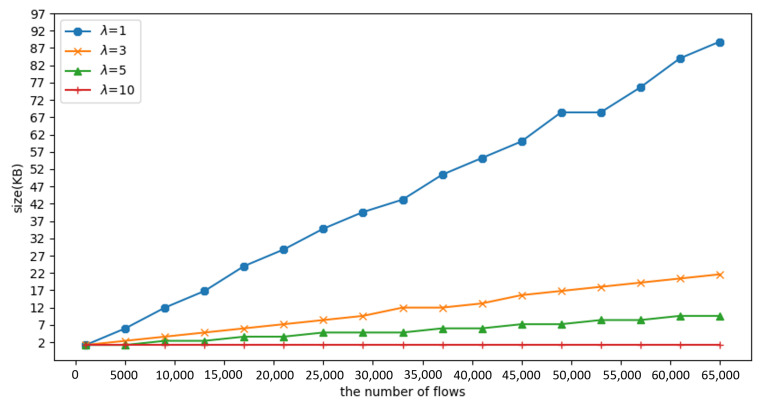
Experiment 5: The required size of register with different λ.

Figure 11 shows the size of the required registers for different λ values. Since the method proposed in this paper is based on the threshold (*T*) to detect the attackers, if λ is smaller, it means that the item exists in the hash table for a longer time, so more registers are needed to defend all attackers within 60 s of the experimental time.

Based on the experimental results, we can see that the required register size for λ=1 is about three times larger than for λ=3. It is estimated that the method proposed in this paper requires the same register size as the Bloom filter method at λ=0.05. This means that the attacker sends a SYN packet every 20 s; however, in reality, the attacker will not send packets that slowly during the attack. Therefore, the method proposed in this paper still has an advantage in terms of register size.

## 5. Conclusions and Future Work

### 5.1. Conclusions

This paper proposes a method to detect and mitigate SYN flooding in P4 switches, which consists of designing a data structure to store the values in the registers and detecting the attacker by calculating the SYN/ACK packets. Then, we take advantage of the P4 switch to send the information to the controller when an attacker is detected and record the MAC address in the blacklist or whitelist. Finally, we perform the implementation in the bmv2 P4 software switch and conduct experiments.

In the experiment, it is found that the percentage of malicious traffic is reduced by half compared with TCP reset. Compared with the Bloom filter, it can improve the accuracy by 2% and save half of the registers.

### 5.2. Future Work

Adding blacklists and whitelists consumes additional space and requires a free timeout for each item in the parameter. If an item in the table has not been accessed for a while, the switch will delete the item. However, if the idle time set is too short, it will cause the controller to update the content frequently. Therefore, an appropriate idle timeout parameter should be set in the future to save space in the table and to avoid frequent updates that can cause a large increase in load between the controller and the switch.

In the future, it is expected that the method proposed in this paper can be implemented more effectively by setting the appropriate idle timeout parameter for each item with a real dataset of SYN flooding, or by setting the parameter automatically in a dynamic manner. It may also be possible to estimate the size of the registers required to stop all attackers within a given time by deriving an applicable equation. In the future, it is expected to be implemented on real P4 hardware (e.g., switch with Intel Tofino chip) to verify the method proposed in this paper.

## Data Availability

No new data were created or analyzed in this study. Data sharing is not applicable to this article. Since our data is generated by simulation, it lacks the need to be shared.
